# Utilizing historical maps in identification of long-term land use and land cover changes

**DOI:** 10.1007/s13280-023-01838-z

**Published:** 2023-02-25

**Authors:** Janne Mäyrä, Sonja Kivinen, Sarita Keski-Saari, Laura Poikolainen, Timo Kumpula

**Affiliations:** 1 Quality of information, Finnish Environment Institute (Syke), Latokartanonkaari 11, Helsinki, 00790 Finland; 2 Department of Geographical and Historical Studies, University of Eastern Finland, Yliopistonkatu 7, Joensuu, 80101 Finland; 3 Department of Environmental and Biological Sciences, University of Eastern Finland, Yliopistonkatu 7, Joensuu, 80101 Finland

**Keywords:** Historical maps, Land use and land cover change, Semantic segmentation, U-net

## Abstract

Knowledge in the magnitude and historical trends in land use and land cover (LULC) is needed to understand the changing status of the key elements of the landscape and to better target management efforts. However, this information is not easily available before the start of satellite campaign missions. Scanned historical maps are a valuable but underused source of LULC information. As a case study, we used U-Net to automatically extract fields, mires, roads, watercourses, and water bodies from scanned historical maps, dated 1965, 1984 and 1985 for our 900 km$^2$ study area in Southern Finland. We then used these data, along with the topographic databases from 2005 and 2022, to quantify the LULC changes for the past 57 years. For example, the total area of fields decreased by around 27 km$^2$, and the total length of watercourses increased by around 2250 km in our study area.

## Introduction

Anthropogenic land change is a significant threat to natural habitats and ecosystem functioning. In European boreal forests, major land cover transitions and following ecosystem disturbances arise mainly from transformation of forests to other land cover types (Ruckstuhl et al. 2008). In addition to the major areal land changes, intensification of human activities also increases linear infrastructure, such as roads, ditches, and powerlines in the landscapes. These features have numerous direct and indirect impacts on the surrounding ecosystems through, for example, habitat fragmentation, alteration of biophysical properties of environment, edge effects, and general increase of other human disturbances (Trombulak and Frissell 2000; Benítez-López et al. 2010).

To quantify and understand the extent and magnitude of environmental changes, robust information of both current and past conditions is needed (Barnosky et al. 2012; Zu Ermgassen et al. 2012). In practice, the baseline, or the starting point of comparisons, is set on the basis of available data (Börjeson 2009). Areal land cover changes of forests, due to drainage of mires or transition from arable land, can be efficiently mapped using worldwide satellite remote sensing techniques over the past three to four decades (Hansen et al. 2013; Belward and Skøien 2015), and historical aerial photographs provide information even from earlier decades. However, the coarse spatial resolution of old satellite images and the inability to detect features below the canopy layer in satellite and aerial images limit their utilization in mapping linear features (Bhattacharjee et al. 2021).

The availability of digital historical maps has increased, which provides new opportunities to study land use changes over various time scales (Fuchs et al. 2015; Kaim et al. 2016). For example, Bičík et al. (2001) examined major land use changes and their drivers over 150 years using old cadastral maps; Skaloš et al. (2011) used old military survey maps and orthophotograph maps to analyze long-term land cover dynamics in the Czech Republic; and Cousins et al. (2015) utilized old cadastral maps to study land cover changes in southern Sweden during the 20th century. Long-term studies on the evolution of linear features in the landscape are generally scarce (Uhl et al. 2022). As a rare example, Bürgi et al. (2015) analyzed road and drainage networks in Swiss lowlands based on historical maps.

There has been a wide array of methods used to extract information from digitized historical maps, ranging from simple color clustering to morphological operations, k-means clustering and, most recently, deep learning methods (Jiao et al. 2021). For instance, Chiang and Knoblock (2010) extracted road pixels based on their color and used k-means clustering to merge similar colors into the same classes. Uhl et al. (2022) utilized recent road network data and compared them with historical maps, outputting the current road segments that most likely existed in the historical maps. In the past few years, U-Net (Ronneberger et al. 2015), a deep learning architecture originally developed for medical image segmentation, has been widely adapted to other domains, including processing and vectorization of historical maps. For instance Ekim et al. (2021) used it to segment various road types from World War II maps; Petitpierre et al. (2021) used U-Net to segment historical maps from two different map corpora; and in the IDCAR 2021 Competition of Historical Map Segmentation, U-Net-based solutions were used by the winning teams for building block extraction and map content segmentation (Chazalon et al. 2021).

In this work, we utilized U-Net to derive land use and land cover information from old, printed maps and created a time series from 1965 to 2022 for our study area in southern Finland. These data were then used to evaluate the changes in the land cover of fields, mires, and water bodies as well as networks of motorways and watercourses. We evaluated the suitability of deep learning-based workflow in obtaining long-term information on land use and land cover changes. Based on the findings, we assessed how to apply this methodology in analyzing landscape changes from old historical maps.

## Materials and methods

### Study area and materials

**Fig. 1:**
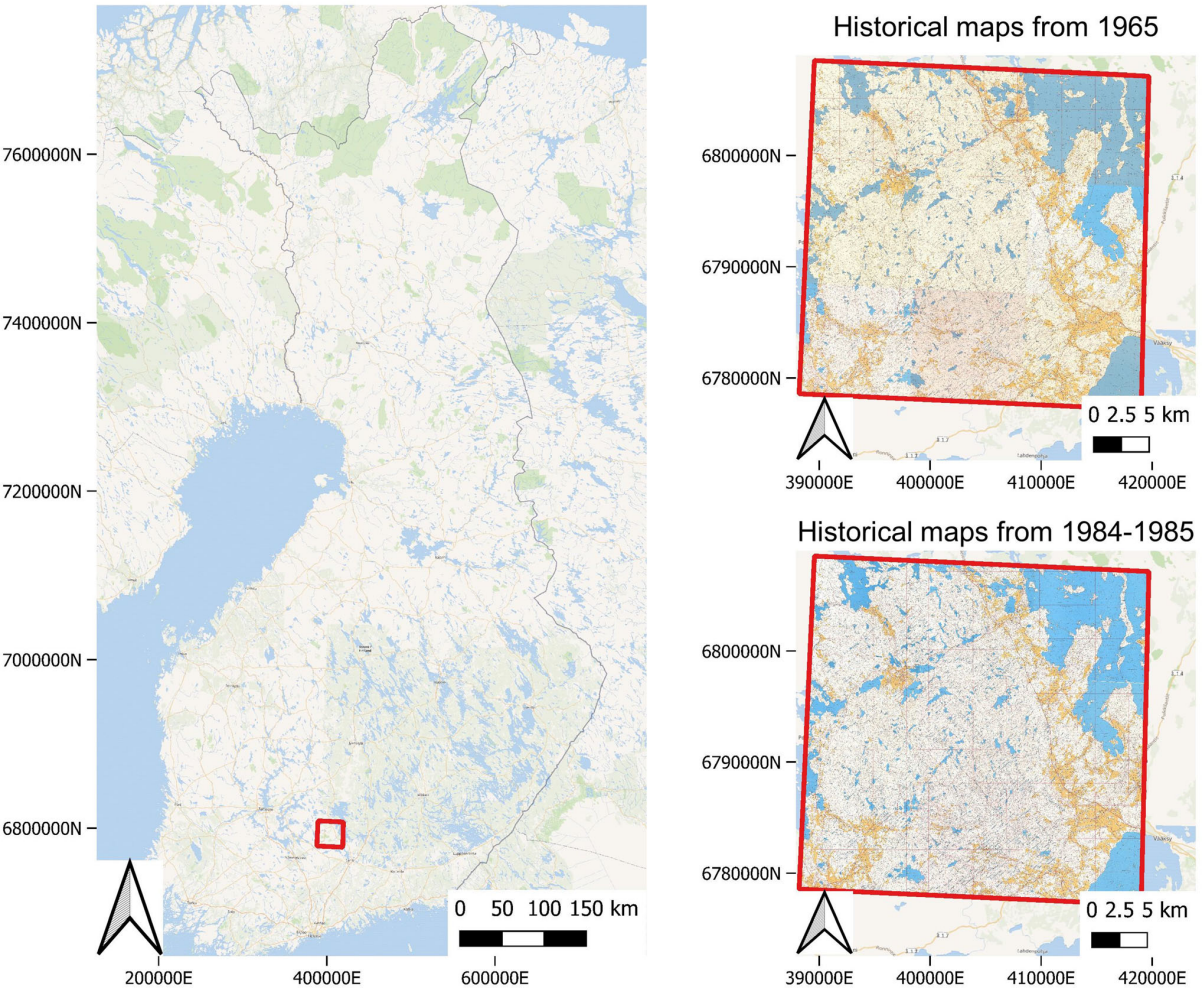
The location of the study area in Finland and the basic maps from 1965 and 1984–1985 covering the area

The study area consists of nine map sheets, each covering an area of $10 \times 10$ km. The study area covers altogether 900 km$^2$ in Evo, Hämeenlinna, Southern Finland (Fig. 1). The area represents a southern boreal forest type, including valuable protected old-growth forests and commercial forest areas, also included in the proposal as Evo Science National Park. Historical maps used in this work were acquired from the National Land Survey of Finland (NLS), which provide around 11 000 scanned basic map sheets with the scale of 1:20 000, originally published between 1949 and 1998. For our study area, the oldest available basic map sheets to cover the whole area were from 1965. For the 1980s, six of the map sheets were originally printed in 1984, and three were printed in 1985. The ground control points for georeferencing the maps were acquired from https://vanhatkartat.fi by Shingle Oy. For the land use and land cover data from 2005 and 2022, we used the topographical database of Finland by the NLS. After georeferencing, the spatial resolution of the maps was around 1.7 m per pixel.

**Fig. 2:**
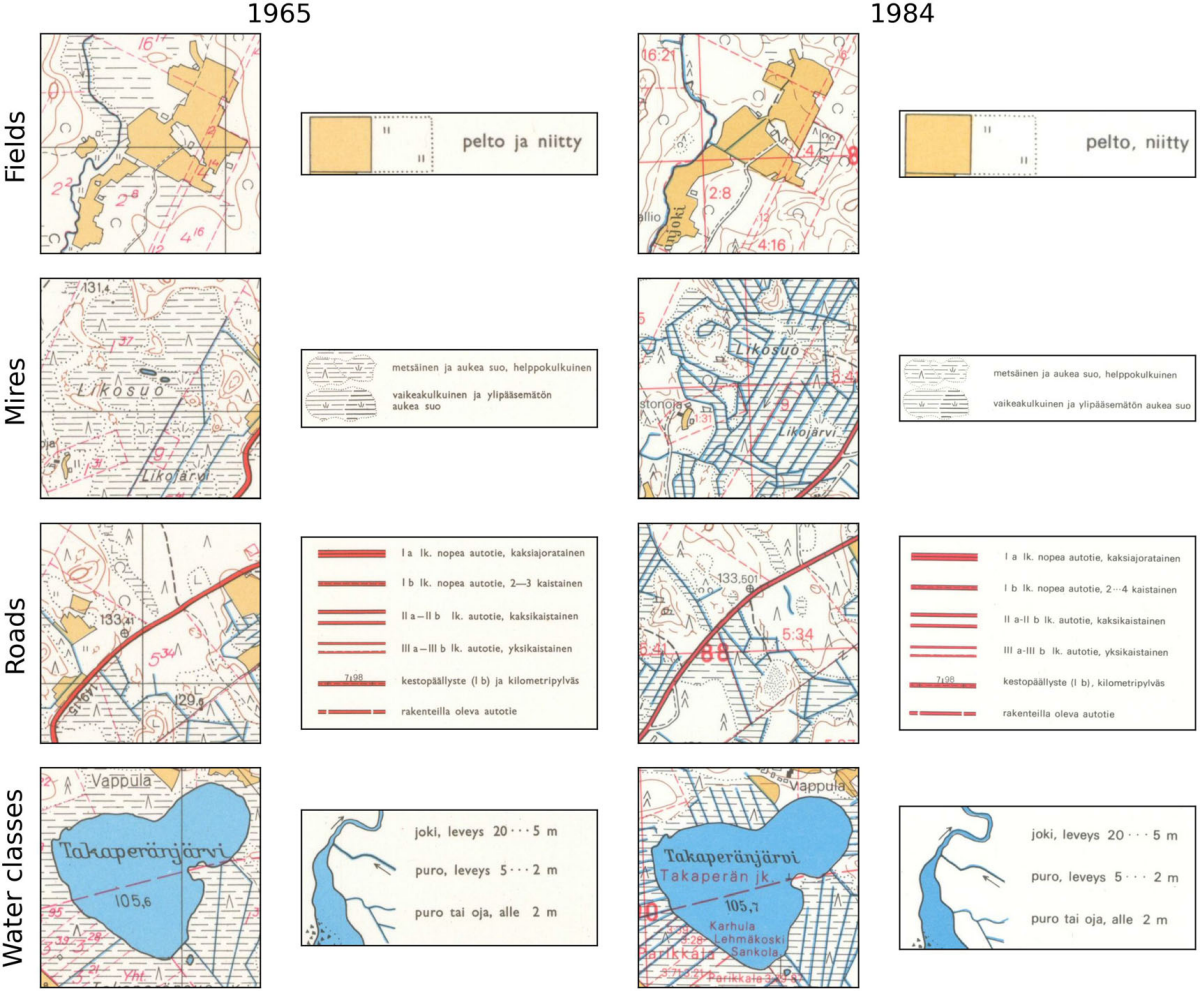
Legends for fields, mires, roads, and water bodies in the historical maps, along with example map scenes containing them. Class “Niitty” (Meadows) was not included in our analyzes. There was no separate legend symbol for water bodies

We manually annotated all the classes of interest from both 1965 and 1984 map sheets with ID 213405 (the south-east corner of our study area) as raster data to use as reference data. In this study, we used five target classes to demonstrate what kind of information is possible to extract from the historical maps: “Fields”, “Mires”, “Roads”, “Watercourses”, and ”Water bodies” (Table 1; Fig. 2). Some of these classes, such as “Mires” and “Roads” contained multiple related subclasses that had slightly different markings but were similar for our purposes. “Mires” contained the classes from the topographical database that have a peat layer of 0.3 m or more, which excludes the subclass “Paludified area” from these analyzes. “Roads” consisted of the road types that have a width of more than 3 m that are marked with wide red lines on the maps. “Watercourses” did not contain the roadside ditches, as they were not included in any of the reference databases.

**Table 1:** Derivation of the target classes for automatic segmentation, along with their equivalents in topographical database

Target class	Basic maps	Topographical database
Fields	Arable land	Field
Mires	Marsh, easy to traverse, open	Open bog, easy to traverse, treeless
	Marsh, easy to traverse, forested	Bog, easy to traverse, forested
	Marsh, difficult to traverse, open	Open fen, difficult to traverse, treeless
	Marsh, insurmountable, open	Fen, difficult to traverse, forested
Roads	Ia class motorway	Motorway Ia
	Ib class motorway	Motorway Ib
	IIa–IIb class motorway	Motorway IIa
	IIIa–IIIb class motorway	Motorway IIb
	Motorway under construction	Motorway IIIa
		Motorway IIIb
Watercourses	River, width 20–5 m	Watercourse area
	Brook, width 5–2 m	Watercourse, 2–5 m
	Brook or ditch, width under 2 m	Watercourse, width under 2 m
Water bodies	Water area	Lake water

### Pre-processing steps

**Fig. 3:**
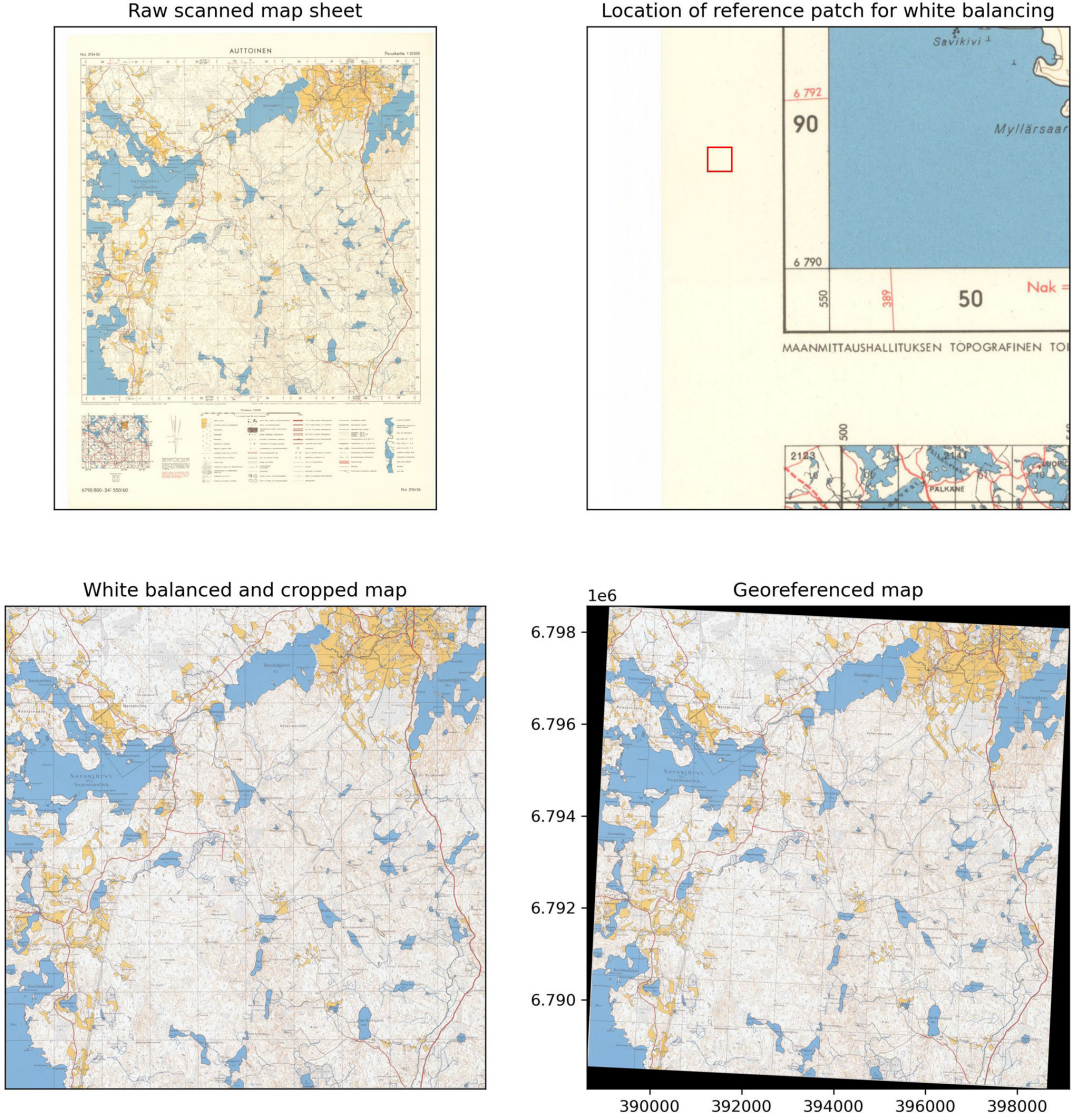
Different steps of basic map pre-processing

As the maps contained a lot of redundant information, such as ticks for coordinates and a legend for map symbols, the first step was to crop the images to only contain the relevant information. Also, to ensure that the each map sheet had similar color balance, we adjusted the white balance of the map sheets based on the white reference patch extracted from the edge of the map sheet. These color balance differences were especially seen from the 1965 maps in Fig. 1, as some of the maps had either yellow or reddish tint compared to the well-preserved maps. The colors were adjusted with the following equation:

1 $$\begin{aligned} {\text {Luminance}} &= \frac{(R_{\text {ref}} + G_{\text {ref}} + B_{\text {ref}})}{3}\\ \nonumber R &= \frac{R_{\text {im}} \times {\text {luminance}}}{R_{\text {ref}}} \\ \nonumber G &= \frac{G_{\text {im}} \times {\text {luminance}}}{G_{\text {ref}}} \\ \nonumber B &= \frac{B_{\text {im}} \times {\text {luminance}}}{B_{\text {ref}}} \end{aligned}$$

where $R_{\text {ref}}$, $G_{\text {ref}}$, and $B_{\text {ref}}$ are the mean color values for each color band of the reference white patch, and $R_{\text {im}}$, $G_{\text {im}}$, and $B_{\text {im}}$ are the color bands of the unprocessed map sheet. The final processed image was constructed from the *R*, *G*, and *B* values, clipped between 0 and 255, and converted to unsigned integer. The different steps of the pre-processing chain are shown in Fig. 3.

We evaluated the scanning and rectification errors for the map sheets by scattering 20 control points on each of the map sheets, using corners of the parcel boundaries as the reference locations. As the reference locations, we used forest stand boundaries from 2022. One of the map sheets from 1965 did not contain the boundary information, so it was omitted from dislocation error analyzes. We then calculated the deviation from 2022 data by the Root Mean Squared Error (RMSE), so that

2 $$\begin{aligned} RMSE = \sqrt{\frac{1}{n} \sum _1^n x_{i}^2 + y_{i}^2} \end{aligned}$$

where *n* is the number of control points (160 for 1965 maps, 180 for 1980s maps), and $x_i$ and $y_i$ are the residuals compared to 2022 data. The maps from 1965 had an RMSE of 12.01 m and the maps from the 1980s had an RMSE of 10.8 m.

### Segmentation methods

The simplest way to extract information from old map sheets is by selecting areas with certain color and using them as masks for land cover classes, such as light orange for fields and blue for water. The main advantage of this method is that it does not require any training data, as the color thresholds are determined by humans, and it has light computation costs. Of course, this method requires annotated validation data to evaluate the results and annotation takes time. However, this type of approach has difficulties with uncommon situations. For example, brooks and ditches running through fields remain undetected by segmenting blue areas because they appear green or brown. Moreover, some colors can mark multiple unrelated classes, like red, which is used to mark both motorways and municipality borders. Finally, some land cover classes, such as mires, are marked with texture instead of color, making them impossible to find by only extracting colors.

We used U-Net (Ronneberger et al. 2015), with pretrained ResNet152 as the backbone for our encoder as our segmentation model. For data augmentation, we used random brightness, contrast and saturation modifications, random horizontal flips, random rotations with a maximum of 5 degrees, and random erasing. We used fastai library version 2.7.8. (Howard and Gugger 2020) and PyTorch version 1.10.1 (Paszke et al. 2019) to train the model, using a single Nvidia V100 GPGPU with 32GB of RAM. The model was trained for 1 frozen epoch and 10 unfrozen epochs, with Adam optimizer and a maximum learning rate of 0.0001 with one cycle scheduling.

**Fig. 4:**
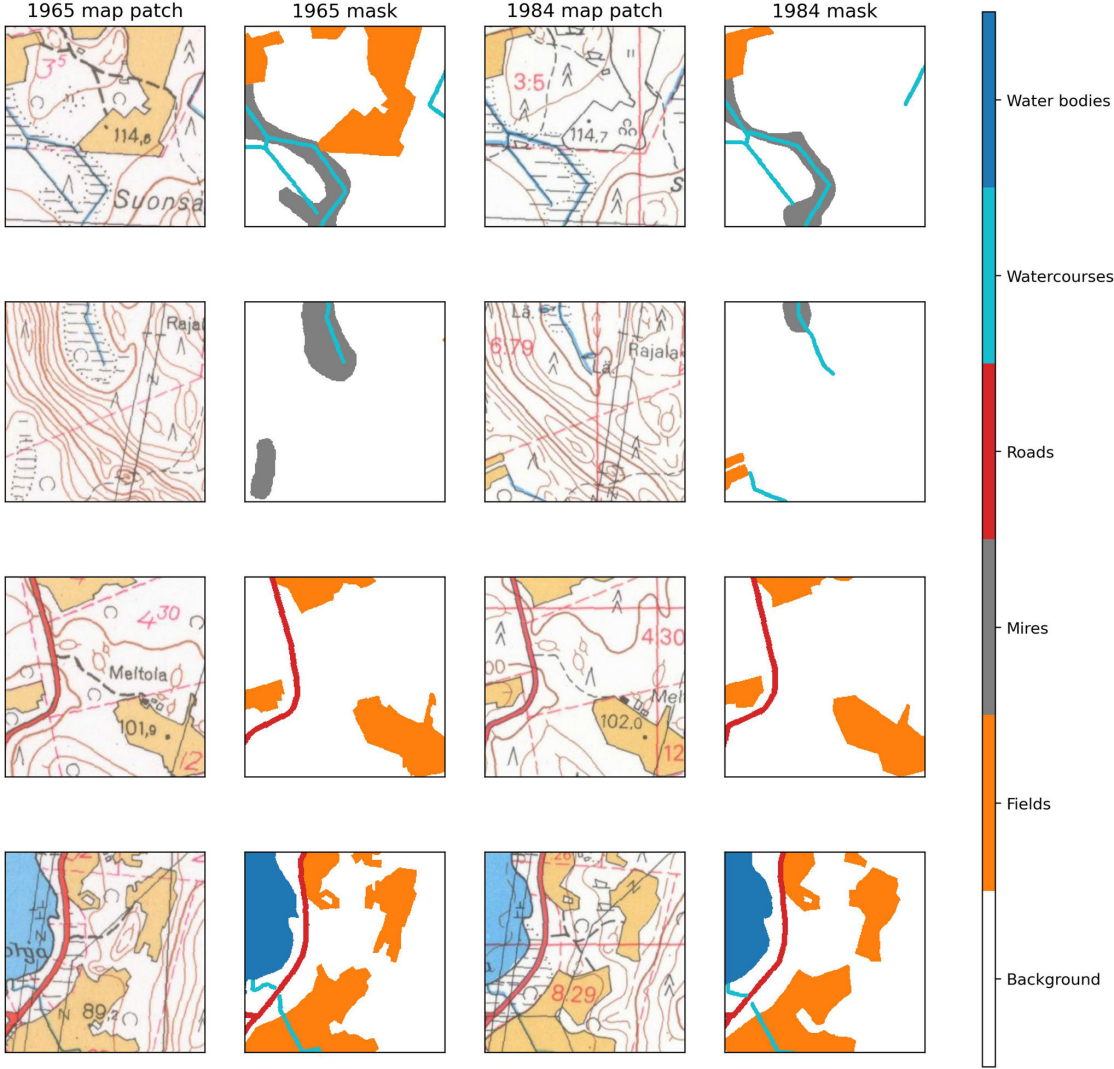
Example $256 \times 256$ pixel (around $435 \times 435$ m) patches from training data

We split our reference data so that the training data contained 75% of the map data and test data contained the remaining 25%. Both map sheets (1965 and 1984) were split so that the training and testing data contain the same geographical area. Also, during the training, we used 25% of the training data as the validation data to monitor the training process. These data were also split such a way that no tiles from validation or test data sets overlapped with the training data set. All data were tiled into $256 \times 256$ pixel tiles without overlap, so that the training data set contained 576 tiles, the validation set contained 192 tiles, and the test set contained 384 tiles. Examples of training data are shown in Fig. 4.

We used Focal loss (Lin et al. 2018) as the loss function for our model, and Precision (Pre), Recall (Rec), and multi-class versions of Dice and Jaccard coefficients as the evaluation metrics. Precision is the ratio between correctly predicted instances and all predictions, whereas recall is the ratio between true positives and all ground truths. Dice coefficient, also known as F1-score, is defined as twice the area of overlap between predictions and targets, divided by the total number of pixels, whereas the Jaccard coefficient, also known as IoU, is the area of intersection between predictions and targets, divided by the area of union. Both metrics were computed for each class separately, excluding the background class, and then averaged with the number of classes (macro averages).

All analyzes and the full workflow for this study are available on https://github.com/mayrajeo/historical-maps.

### Post-processing steps for full map tiles

While producing the land cover maps for all of the full map sheets, we mosaicked them into $256 \times 256$ pixel tiles with a 128 pixel overlap, and afterwards de-mosaicked the predictions to cover the full map sheet. As the models made most of the mistakes near the edges of the tile, we discarded the predictions near the edges so that each $256 \times 256$ pixel tile produced a $192 \times 192$ pixel prediction mask. This step resulted in a single band raster for each map sheet, where each pixel had a value between 0 and 5 corresponding to a single class, with 0 being a background pixel.

In order to improve the quality of predictions and reduce noise, such as singular pixels of a single class scattered or remnants of letters within the predictions, we applied a set of morphological operations for each class separately. For water bodies, we ran a morphological opening (erosion followed by dilation) with an $11 \times 11$ kernel and low-pass filter with a $7 \times 7$ kernel. For roads and watercourses, we applied a morphological closing (dilation followed by erosion) with a $7 \times 7$ kernel and low-pass filter with a $5 \times 5$ kernel. Because fields and mires should not be split by a brook or a ditch into several smaller segments, field and mire predictions first included watercourses as well. Watercourses outside fields or mires were eliminated by eroding the layers with an $11 \times 11$ cross shaped kernel, which was then followed by dilation with an $11 \times 11$ rectangular kernel and a low-pass filter with a $7 \times 7$ kernel. Finally, fields and mires were clipped with processed roads and water bodies. The final result after post-processing was a five-band raster with a spatial resolution of around 1.7 m, where each band corresponded to a binary mask for a single class.

The final post-processing step was to convert roads and watercourses into line geometries. The post-processed raster data for these classes were first skeletonized into one-pixel-wide representations and then converted into polygons. These polygons were then buffered with 10 m, all intersecting polygons were merged together, and the resulting polygons were eroded with 10 m. Finally, we used centerline library (Todic 2022) to convert these polygons into line geometries.

### Evaluating the land use and land cover change

As the georeferenced map sheets were not perfectly aligned, we examined the changes in land use and land cover by aggregating the data from different time periods into a $500 \times 500$ m regular grid and computed the changes based on total area or total length within the grid cell. This was done to make it easier to find the areas with the most drastic changes and to mitigate the slight location errors between the data from different years and sources. In addition, for watercourses, we derived the total length within mires and fields in order to better analyze the change types.

## Results

### Model evaluation

**Fig. 5:**
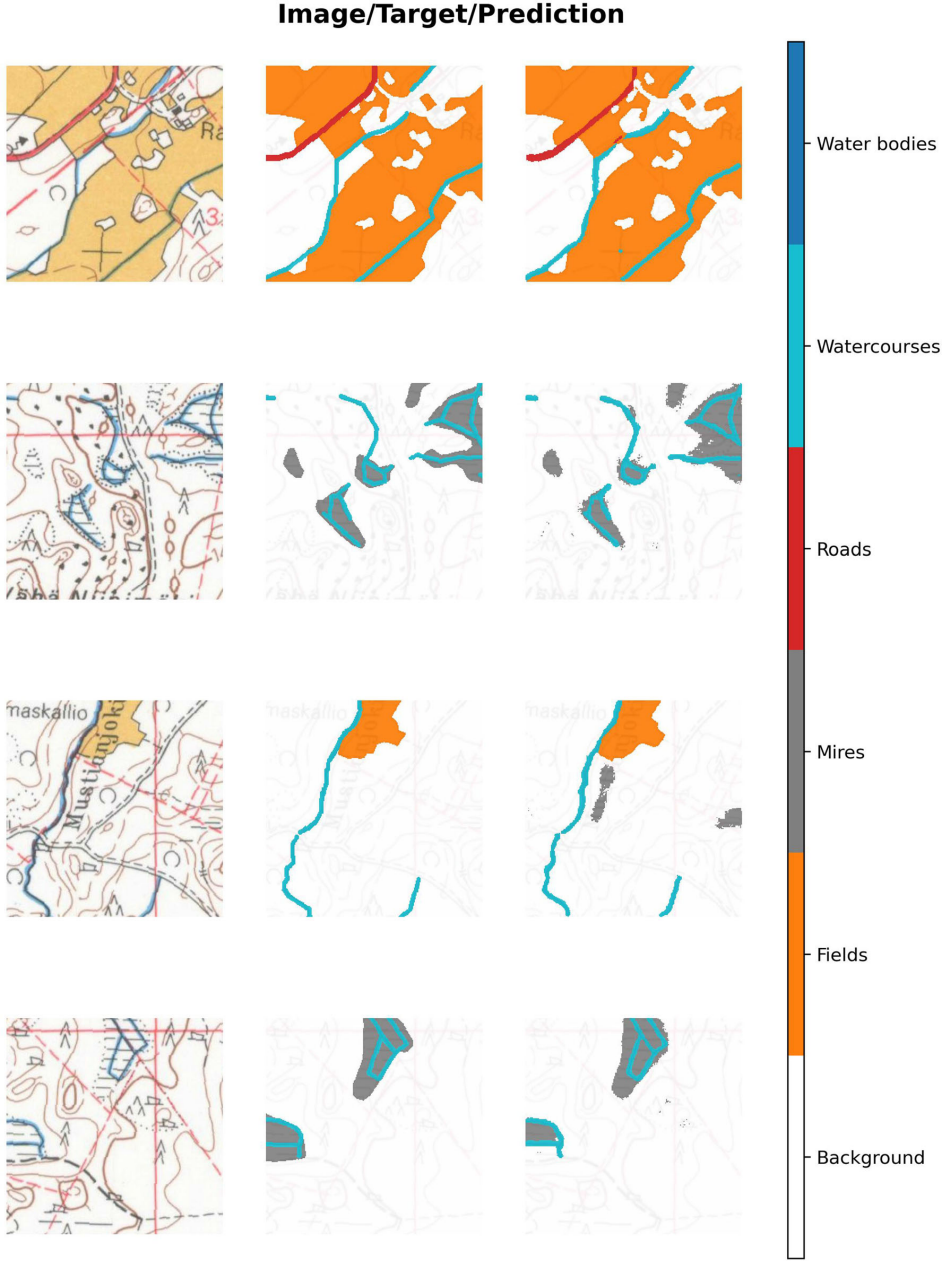
Example predictions from the test set for $256 \times 256$ pixel (around $435 \times 435$ m) patches

Our model achieved excellent results (Table 2), as the overall Dice score for the test set was around 0.92 and the Jaccard score around 0.83. The easiest classes to predict were fields and water bodies, which both had near-perfect results. Watercourses were the most difficult class to segment, as in some cases they were undetected when passing through either fields or mires, and in other cases, dark lines, such place-name letters, were misidentified as watercourses (Fig. 5). The most common error-prone situations for the model were those in which either a road or a watercourse was surrounded by fields, and those when paludified areas were misidentified as mires when located next to each other. Mires went also unidentified by the model in the pixels where place-name, numbers, or forest type markings prevented the occurrence of mire markings (Fig. 5). In some cases, the model did not detect the dotted lines that mark the shape of the mire area and inflated these areas.

**Table 2:** Validation and test set results for land cover predictions, for $256 \times 256$ pixel image patches

	Validation				Test			
	Pre	Rec	F1	IoU	Pre	Rec	F1	IoU
Fields	0.972	0.974	0.973	0.948	0.962	0.978	0.970	0.941
Mires	0.884	0.898	0.891	0.804	0.872	0.925	0.898	0.815
Roads	0.853	0.902	0.877	0.781	0.846	0.920	0.882	0.788
Watercourses	0.747	0.799	0.772	0.629	0.783	0.819	0.801	0.668
Water bodies	0.991	0.995	0.993	0.986	0.987	0.992	0.989	0.979
Overall	0.890	0.914	0.915	0.829	0.890	0.927	0.920	0.838

From the patch predictions before the post-processing chain, it could be seen that the predicted fields and mires were blurred and there were less distinct edges than in the target masks; and overall, the predictions were most inaccurate near the edges of each image. These factors showed the need for a post-processing chain to discard the edge predictions. Results for the full test area are presented in Table 3, both before and after the post-processing chain. The most notable differences between image patches and the full area can be seen for mires and waterways, as both metrics are clearly lower for collated predictions, both before and after post-processing. Roads benefited the most from the post-processing chain, as their Dice and Jaccard scores improved by 0.037 and 0.054 respectively; the only class with no improvement was fields. Overall, results for the 1965 map sheet were slightly better than for 1984.

**Table 3:** Full area results for 30 km$^2$ test set before and after post-processing

		Fields	Mires	Roads	Watercourses	Water bodies	Overall
Before post-processing							
1965	Pre	0.928	0.827	0.832	0.713	0.987	0.857
	Rec	0.954	0.902	0.830	0.706	0.988	0.876
	F1	0.941	0.863	0.831	0.709	0.987	0.866
	IoU	0.888	0.759	0.711	0.550	0.988	0.777
1984	Pre	0.931	0.767	0.734	0.673	0.980	0.817
	Rec	0.952	0.843	0.830	0.703	0.988	0.863
	F1	0.941	0.803	0.779	0.688	0.984	0.839
	IoU	0.889	0.671	0.638	0.524	0.969	0.738
All	Pre	0.929	0.797	0.783	0.693	0.984	0.837
	Rec	0.953	0.873	0.830	0.704	0.988	0.870
	F1	0.941	0.833	0.805	0.699	0.986	0.853
	IoU	0.889	0.715	0.674	0.537	0.972	0.757
After post-processing							
1965	Pre	0.909	0.867	0.817	0.691	0.992	0.855
	Rec	0.955	0.889	0.926	0.832	0.983	0.917
	F1	0.931	0.878	0.868	0.755	0.988	0.884
	IoU	0.872	0.783	0.767	0.607	0.975	0.801
1984	Pre	0.914	0.835	0.718	0.650	0.986	0.821
	Rec	0.955	0.810	0.945	0.864	0.984	0.911
	F1	0.934	0.823	0.816	0.742	0.985	0.860
	IoU	0.876	0.699	0.689	0.590	0.971	0.765
All	Pre	0.912	0.851	0.767	0.671	0.989	0.838
	Rec	0.955	0.850	0.935	0.848	0.984	0.914
	F1	0.933	0.842	0.842	0.749	0.986	0.872
	IoU	0.874	0.728	0.728	0.598	0.973	0.783

We also computed the error of the total areas and lengths for the test set. For fields, the total area based on predictions was almost equal, with 0.03 km$^2$ (1.4 % of total annotated area) less fields predicted for 1965 maps and 0.02 km$^2$ (1.1 %) less for 1984 maps. For mires, the differences were also small: 0.129 km$^2$ (2.2 %) less for 1965 maps and 0.2 km$^2$ (3.3 %) less for 1984 maps. The predictions underestimated the total length of roads by 0.15 km (0.9 %) for 1965 maps and by 0.63 km (3.9 %) for 1984 maps. Watercourses were the only class where the predictions overestimated the amount for both years, as the total length was overestimated by 3.5 km (5.8 %) for 1965 maps and 4.3 km (2.6 %) for 1984 maps. Water bodies were predicted almost perfectly, as the total area was 0.01 km$^2$ (0.4 %) more for 1965 maps and almost equal (0.07 % less) for 1984 maps.

### Land use and land cover change in the study area

The arable land area clearly changed during the study period, as the total area classified as fields decreased from 98.92 km$^2$ in 1965 to 71.75 km$^2$ in 2022 (Table 4). The total area classified as mires fluctuated with a gain of about 10 km$^2$ between 1965 and 1985, a loss of about 7 km$^2$ between 1985 and 2005, and a gain of 7 km$^2$ between 2005 and 2022. Local fluctuations in mire areas were even greater than that, with appearances and disappearances of the mires within the 20 year intervals between the mappings.

The total length of motorways increased about 90 km during the observed 57 years. In particular, the increase was highest between 1965 and 1985. Towards 2022, the increase in motorway network was restrained. The most drastic change during the study period occurred for watercourses between 1965 and 1985, as their total length more than doubled, with a gain of 1385 km in that time frame. Nowadays, the total length of watercourses is around three times what it was in 1965. Most of this increase occurred in mires, where the total length of watercourses quadrupled between 1965 and 2022. In contrast, in fields, the total length of watercourses clearly decreased between 1985 and 2005, and still more between 2005 and 2022. Water bodies remained more or less the same during the whole study period, with spatially limited small-scale changes.

**Table 4:** Summary of land use and land cover changes for the study area

	1965	1985	2005	2022
Fields				
Total area	98.92 km$^2$	87.04 km$^2$	75.45 km$^2$	71.75 km$^2$
Gain	–	2.32 km$^2$	1.27 km$^2$	1.81 km$^2$
Loss	–	14.20 km$^2$	12.85 km$^2$	5.51 km$^2$
Change	–	$-$11.87 km$^2$	$-$11.57 km$^2$	$-$3.70 km$^2$
Mires				
Total area	80.91 km$^2$	90.83 km$^2$	83.82 km$^2$	90.71 km$^2$
Gain	–	15.86 km$^2$	1.74 km$^2$	8.04 km$^2$
Loss	–	5.92 km$^2$	8.72 km$^2$	1.14 km$^2$
Change	–	9.95 km$^2$	$-$6.98 km$^2$	6.89 km$^2$
Roads				
Total length	355.27 km	399.26 km	435.57 km	444.13 km
Change	–	43.99 km	36.31 km	8.56 km
Watercourses				
Total length	1168.24 km	2553.05 km	3147.48 km	3321.15 km
Gain	–	1473.98 km	689.11 km	281.52 km
Loss	–	89.17 km	94.68 km	107.85 km
Change	–	1384.81 km	594.43 km	173.67 km
Total length in fields	266.62 km	273.78 km	194.06 km	166.73 km
Total length in mires	447.12 km	1604.50 km	1925.36 km	2104.92 km
Water bodies				
Total area	170.78 km$^2$	169.78 km$^2$	171.01 km$^2$	171.21 km$^2$

**Fig. 6:**
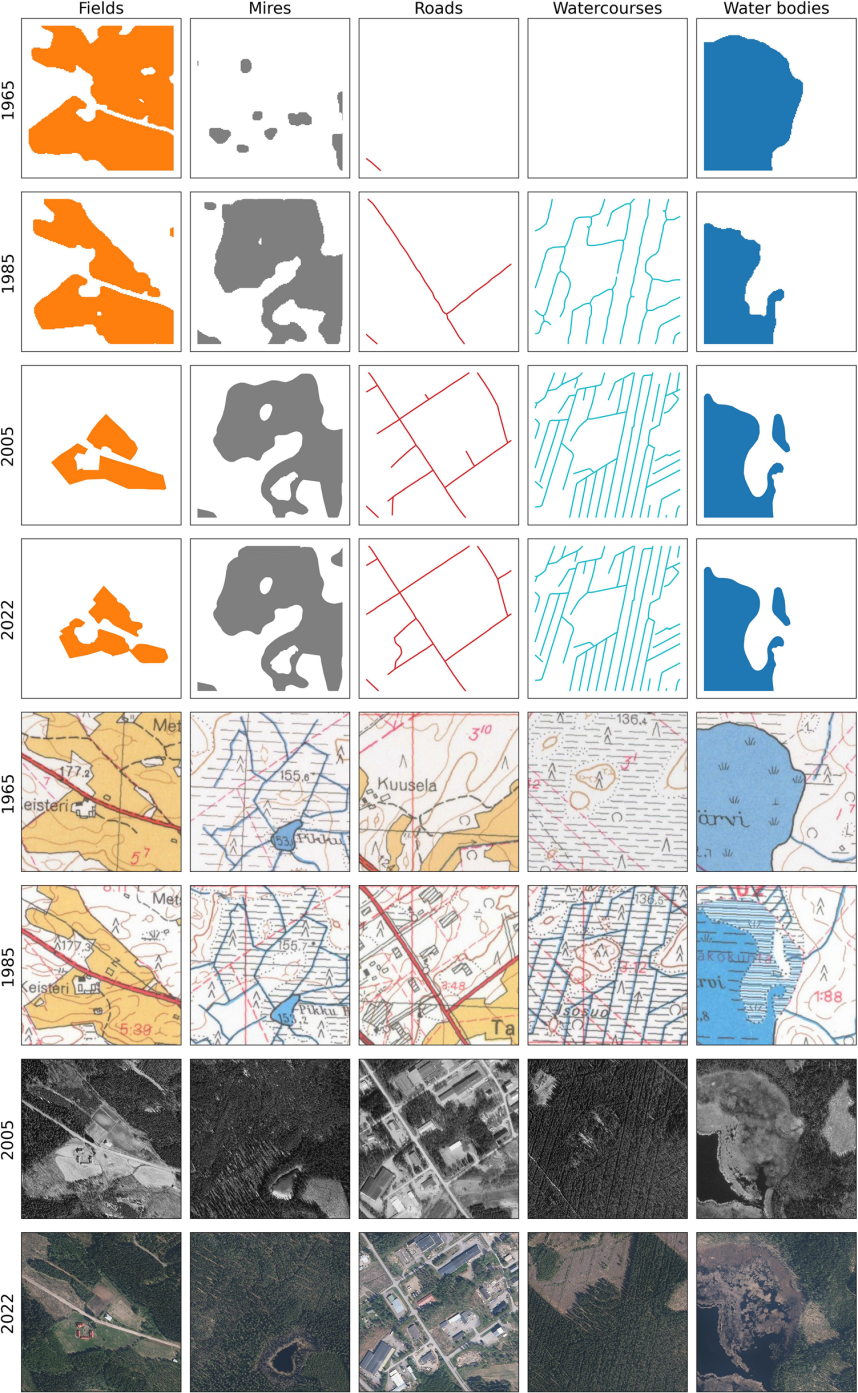
$500 \times 500$ m patches with the most drastic changes for each land cover class

In addition to acquiring the extent of overall changes for our study area, our results enabled detecting and positioning significant local changes between 1965 and 2022. For example, Fig. 6 shows the $500 \times 500$ m grid cells with the most extreme changes from 1965 to 2022 for each class, along with the corresponding map patches or aerial images from the archives of NLS Finland.

## Discussion

Historical maps offered an opportunity to explore the land use and land cover development in the study area since 1965, whereas currently the earliest comparison point for Finland is usually in the early 2000s (Corine Land Cover 2000 or the earliest available topographical database). All maps utilized here were produced using the same mapping methods and the level of detail, which provides a robust baseline for comparisons. The methodological approach used in this study showed high accuracy for analyzing fields and water bodies, and was highly promising for mires, motorways, and watercourses. The map symbols and color palettes employed in historical maps affect the automatized and manual class identification. The symbol system differs among regions, countries, and years. Thus, a single method is not likely to be applicable across different mapping systems, used in different countries or through years, but the methodological principles remain the same. The feature classes in this study were selected to include those for which maps can be considered a reliable source of information and some more difficult ones. Arable land is a land use class with consistent presentation in Finnish basic maps (Vuorela et al. 2002), water bodies represent consistent single-level hydrology features, and motorways marked in red are also easily distinguished in the maps. Watercourses marked in blue and mires outlined with dotted lines represented challenges that we wanted to tackle using an automated method.

### Performance of the proposed method

In this study, U-Net proved to be a suitable method for processing historical maps, achieving an overall F1-score of 0.872, and an overall IoU of 0.783. The differences between the predicted total areas and lengths were minor, with the largest error being around 5 percentages for watercourses in 1984 maps. The most accurately segmented classes were those that cover larger areas (fields, mires, and water bodies), and the linear classes had slightly worse segmentation results. This was expected, because segmentation metrics were measured based on the overlap between predictions and annotations. Especially waterways are narrow and only a few pixels wide, so a small offset between predictions and annotations can have a larger impact on the evaluation metrics compared to classes covering larger areas. U-Net was also able to distinguish between paludified area and mire, where the difference in map markings were for the most part only in the higher or lower density of the horizontal lines. In the future, adding paludified areas as one of the classes might improve the results and usability even further.

One advantage of U-Net over rule-based methods, such as color thresholding or morphological operations, is that given enough training data, U-Net can learn to ignore textures that are not of interest, such as written text, property and municipal boundaries, and height contours. Our model, for the most part, ignored these features, with only minor difficulties with thicker black and red letters, which were classified as waterways and roads respectively. Likewise, it is possible to train a model to detect only these types of features. For instance, Ekim et al. (2021) used a similar U-Net-based method for detecting and classifying different types of road lines from German World War II maps, and classifying past road network from historical Finnish maps is a possible future research topic.

There are, of course, disadvantages of using deep learning methods. The main disadvantage is the amount of required training data and computational costs. Processing maps using morphological operations or color thresholding is fast and does not require heavy computing resources, and the methods do not require external training data, as the parameters are defined by the researchers. Deep learning methods require accurate training data, and creating it is time-consuming. Training the model is also more time-consuming and requires suitable hardware. As for inference time, our model classified one map sheet in around seven minutes, and the most time-consuming part of the presented workflow was actually converting predictions for roads and waterways to line geometries. No matter which method is used, some amount of human annotated validation data is required to validate the results.

### Land use and land cover changes

The clear decrease in arable land area, particularly between 1965 and 1985, is in line with the general trend that occurred in Finland. The area of arable land started to increase rapidly in the end of the 19th century, and this development continued until the 1960s. During the past decades, the afforestation subsidies and the declining number of farms have contributed to the afforestation of set-aside or abandoned agricultural land (Tiainen et al. 2004). The decline of arable land and grasslands in southern Finland during the past decades has been reported, for example by Ruuska and Helenius (1996) and Pitkänen et al. (2014). Countrywide, agricultural land area was 2.7 million ha in the 1960s, with a decrease after that to 2.2 million hectares in 2021 (Natural Resources Institute Finland (Luke) 2022). Likewise, in Norway, the abandonment of agricultural grassland has led into the transition of heathland and grassland into woodland between 1964 and 1989 (Olsson et al. 2000). In the Swiss countryside, historical maps showed an increase in arable land before the 1930s due to the strong promotion of intensive agriculture, and a decrease after that (Bürgi et al. 2015).

Historically, the transformation of mires to agricultural and forestry lands started already in the 1700s in Fennoscandia (Enbuske and Ruuskanen 2021). In Finland, agriculture was the main driver of the draining of mires until the 1950s, and since then, mires have been drained for forestry (Turunen 2008). Forestry drainage aims at increasing timber growth in established waterlogged woodlands, and has also been utilized to enable the afforestation of open or sparsely wooded mires, to promote reforestation on sites experiencing secondary paludification after timber harvesting, and to stabilize forest roads (Lõhmus et al. 2015). In Finland, Estonia, and Sweden, 55, 30, and 14% of the total mire area has been drained for forestry, respectively (Vasander et al. 2003).

The strong increase in watercourses between 1965 and 1985 in the study area, and in whole Finland is a well-known phenomenon due to the intensive drainage contributed by state subsidies, including some regional differences in the intensity of drainage. The rather strong increase in watercourses in the study area, from 1985 to 2005 and even from 2005 to 2022, is surprising, as the peak for draining was generally reached in the 1960s and 1970s (Peltomaa 2007). In contrast to the mire ditching, in fields, the total drainage network length decreased after 1985. The decrease was much more drastic than the one in the total area of fields, which implies that the decrease was mostly due to the shift from open ditches to sub-surface drainage systems. In this study, the watercourses also included natural streams. As the length of the natural streams can be considered stable, this method is well-suited for examining the increase of the length of the drainage network.

The strong fluctuations in the results of the mire area were surprising and are probably related to changes in mapping practices during the 60 years of the study period. The definition of mire has not changed during the years, but in practice, large areas were defined as paludified areas in 1965 and as mires in 1985. This can be due to varying interpretations of the vegetation in the field, misclassifications by the model, and it is likely that some of the paludified areas have turned into mires during the 20 years. The decrease of mire area from 1985 to 2005 may largely be due to a change in vegetation of drained mires to resemble forest vegetation, which has led to their classification as forests in the field survey, as noted by Turunen (2008). The observed increase in mire area between 2005 and 2022 was probably artificial, i.e., classification related, since environmental restoration of mires has hardly been executed in the study area. These changes were not due to our methods, as data from 2005 and 2022 were taken from the topographical database by NLS Finland. Currently, and in the coming years, mire restoration projects are executed in Finland on confined areas. Mire restoration, by filling the ditches, has been shown to successfully enable increased water table and increasing mire vegetation (Maanavilja et al. 2015; Menberu et al. 2016).

The total length of the motorway network increased between every time interval examined, with the biggest increase between 1965 and 1985. The increasing trend in man-made road networks is generally known, but their timings vary between regions. In a case study in Switzerland, in an agricultural lowland landscape, roads were mostly built between 1749 and 1882, and between 1939 and 1943 (Bürgi et al. 2015). In the urban city area of Albany in New York, the number of roads increased more between 1900 and 1930 than between 1930 and 1950 (Uhl et al. 2022). In this study, the roadside ditches on both sides of the roads also play a role in the water drainage system, even though they are not marked as being part of the watercourses, and thus they have an influence on hydrology that is not always recognized.

The decrease in the area of water bodies between 1965 and 1985 can be mostly explained with the decrease of water level in the Tervajärvi lake due to the added drainage system (Fig. 6). Additionally, the model classified some wide rivers as water bodies instead of watercourses. Some minor changes in mapping methods also occurred between 1965 and 1985. Otherwise, the extent of water bodies is high, as typical to Finland.

It is worth noting that there are several potential sources of error when using scanned historical maps for LULC analysis. First, and the most obvious, are the inaccuracies due to survey methods in the past. We observed a slight misalignment between map sheets of different years, which may be due to cartography methods advancing during the study period. The misalignment issues, and the inaccuracies due to this, can be partially addressed by downsampling the resolution to be coarser than the dislocation error (Geri et al. 2010). In this study, we addressed these errors by analyzing land cover changes within $500 \times 500$ m grid cells instead of overlaying the results from different years.

### Impacts on biodiversity and carbon storage

In general, the replacement of arable vegetation by forest vegetation is a slow process, and the outcome depends on the afforestation site, tree species, and forest management, and also the ecological impacts of afforestation depend on site characteristics (Rey Benayas et al. 2007). The information of past agricultural use is important for floodplain forest conservation, as floodplain forests afforested in former arable land do not have as high biodiversity compared to those in a natural state (Brown et al. 1997). Further, information of agricultural land use history can be utilized in evaluating the quality of traditional rural biotopes that significantly contribute to biodiversity in the boreal region (Raatikainen et al. 2017).

Ditching has major impacts on terrestrial and aquatic ecosystems through alterations in soil conditions, hydrology, and tree cover (Holopainen and Lehikoinen 2022). However, drainage is often overlooked as an ecosystem modifier, as it changes the ecosystem slowly and the impacts can be difficult to observe. The impacts of forest drainage on biodiversity and threatened species during the different phases of post-drainage succession are particularly poorly known (Lõhmus et al. 2015).

The effect of drainage-network increase in mires has clearly had consequences on carbon sequestration and storage in mires. Previously, drainage was considered to increase the carbon sink of mire vegetation due to increased growth, even though the uncertainty of the estimates was also acknowledged (Turunen 2008). However, the carbon storage of peat decreases after drainage, and drained mires become carbon sources (Simola et al. 2012). Expanded drainage systems affect the water quality, for example by elevated pH and increased organic nitrogen content (Prévost et al. 1999). On biodiversity, mire drainage has had a strong negative impact due to loss of mire vegetation and increased similarity to forest vegetation (Ojanen et al. 2020). On the other hand, the ditch-network increase in fields, particularly before 1965 and maybe also between 1965 and 1985, had a positive effect on biodiversity, because it created diverse, small-scale traditional farmland habitats, and open ditches were biodiversity-supporting habitats for many plant and insect species (Jauni and Helenius 2008). The development of sub-surface drainage since 1985 has had a negative impact on biodiversity, because the landscape has become more monotonous with the decreasing number of open ditches that has led to growth of field parcels (Hietala-Koivu et al. 2004).

For road networks, our case study included only the motorways with map markings in red. Further analysis should also include the development of forest road network, which is a challenge with the Finnish mapping symbol system due to the black color being used for multiple item classes. From the perspective of biodiversity, the development of forest road networks has an evidently high impact due to the fragmentation effect on forest landscapes. Thus, for accessibility of forest areas, forest road mapping would be needed, whereas the motorway network development analyzed in this study mainly affects the mobility of people.

### Future challenges

Our study area covered 900 km$^2$ to test the workflow, but in many cases, it is reasonable to expand the analyzes to a larger scale in order to support the information needs of the administrative units. For example, for a river catchment level hydrology analysis, the knowledge of watercourse network expansion, both downstream and upstream, is needed (Bhattacharjee et al. 2021). Such knowledge of drainage systems is also of importance for the assessment of maintenance needs or candidate mires for restoration (Hasselquist et al. 2018). To include older maps than 1965 would be of great value, but it also introduces availability and comparability issues, such as geometric distortion, number of ground control points available, differences in landscape categories used, distinctiveness of symbol representation, and thematic consistency of each feature class (Vuorela et al. 2002). In practice, even with 1965 as the oldest source map, the analysis of extended areas with a larger number of feature classes would be highly influential to the understanding of historical land use in Finland.

Workflow development needs to be continued to include more land cover classes besides those in this study. Methodologically, of course, challenges with a higher risk of misinterpretation are created when the number of classes is increased, because of matching colors used for multiple classes and difficulties in outlining the new classes. Particularly forest classes (deciduous, coniferous, and mixed forests) in Finnish maps have ambiguous outlines with no clear border markings. Forest classes would nevertheless be especially of interest for further study. This exercise showed that deep learning methods can highly accurately deal even with relatively vague classes, such as mires.

## Conclusions

In this study, we demonstrated how to utilize modern computer vision methods to derive georeferenced information from scanned historical maps. We used U-Net to detect five classes of interest from scanned basic maps from 1965 and the mid-1980 s, and the proposed method proved to perform well. The results were used to analyze land cover and land use changes in the study area between 1965 and 2022. The analysis showed the increase with road and ditch networks and the change in area of agricultural fields and mires. The observed land cover changes are in line with the known development in forestry and agriculture.

Efficient utilization and digitization of historical maps can greatly improve the knowledge of past land use and land cover changes; as for example, in Finland, the archives of the NLS contain over 10 000 map sheets, dated between 1949 and 1997. In the future, we aim to further develop our method both by increasing the study area and by including more classes.
